# Nontypeable Hemophilus Influenza Meningitis in a Primary Sjögren's Syndrome Patient on Hydroxychloroquine

**DOI:** 10.7759/cureus.39601

**Published:** 2023-05-28

**Authors:** Gizem Yassa, Fahad Khan, Nicholas Manticas, Delaram Majlesi, Farah Zahra

**Affiliations:** 1 Department of Internal Medicine, Chicago Medical School Internal Medicine Residency Program at Northwestern Medicine McHenry Hospital, Mchenry, USA; 2 Department of Internal Medicine, Chicago Medical School Internal Medicine Residency Program at Northwestern Medicine McHenry Hospital, McHenry, USA

**Keywords:** hydroxychloroquine, connective tissue diseases, hemophilus influenza meningitis, primary sjogren’s syndrome, hemophilus influenza in adult

## Abstract

Primary Sjögren's syndrome is a multisystem autoimmune disease that less commonly requires immunosuppression compared to other systemic connective tissue diseases and classically has a poorer correlation with increased incidence of infections. Herein, we describe a 61-year-old female without predisposing factors diagnosed with the uncommon nontypeable* Hemophilus influenza* meningitis complicated by sepsis.

## Introduction

Primary Sjögren's syndrome (pSS) is a multisystem autoimmune disease characterized by lacrimal and salivary gland dysfunctions and extraglandular organ involvements. Compared to other connective tissue diseases, it is reported to have a milder increased infectious complication risk, especially in those who are not using immunosuppressive medications. Nontypeable *Hemophilus influenza* (NTHi) rarely causes invasive disease in the general population. Reported infections commonly had a predisposing factor. Meningitis, in particular, comprised a minuscule percentage of invasive *H. influenza* infections.

We present a case of a pSS patient on hydroxychloroquine (HCQ) who developed community-acquired NTHi meningitis, presumably following otitis media. This report presents a rare form of invasive NTHi infection despite the absence of predisposing conditions and the literature reporting a lower risk of infections in pSS patients, especially in those receiving HCQ and those who are not on any immunosuppressive medications.

## Case presentation

Our patient is a 61-year-old female with a history of hypothyroidism, hypertension, class-II obesity, and pSS diagnosed five years ago with arthralgias, myalgias, fatigue, and dryness of the eyes and mouth who was on HCQ 200 mg twice daily since her diagnosis. She presented due to fever, altered mental status, and vomiting. She had an episode of non-bloody non-bilious vomiting in the morning and was noted to have a fever of 103.3°F, new-onset agitation, confusion, visual hallucinations, inability to engage in conversations, and diaphoresis. The day before, she complained of left earache and headache, which subsided with acetaminophen. At baseline, the patient was alert, fully oriented, independent, and employed at an office job. She had a five-pack-year remote smoking history and no alcohol or drug use. She did not have recent travel history, sore throat, cough, dysuria, abdominal pain, or rashes.

Vitals were remarkable with a heart rate of 101/minute, fever of 103.3°F, respiratory rate of 29/minute, and blood pressure of 149/71. Physical exam was notable for severe agitation, confusion, and uncooperativeness. Kernig's sign and Brudzinski's sign were negative. Heart, lung, and abdominal exams were unremarkable. She had no focal neurologic deficits or nuchal rigidity. Glasgow coma scale score was as follows: eyes = 4, verbal = 3, and motor = 5. Lab results showed that neutrophilic leukocytosis was 19.8 x 10^3^/µL, potassium was 3.1 mmol/L, procalcitonin was 2.156 ng/mL, and lactic acid was 3.9 mmol/L. Urinalysis and urine toxicology were unremarkable.

Computed tomography (CT) of the chest, abdomen, and pelvis showed subtle ground-glass opacities of bilateral upper lobes though she had no cough or respiratory symptoms. CT head and cervical spine were negative for any acute changes. Paranasal sinuses and mastoid air cells were clear. Neurology was consulted and recommended lumbar puncture with a likely need for anesthesia, given the severity of the agitation. Magnetic resonance imaging was unable to be performed due to body habitus. Blood cultures were collected, and she was empirically started on vancomycin, ampicillin, ceftriaxone, acyclovir, and dexamethasone. After sedation, she was noted to have a bulging left tympanic membrane without discharge. Ceftriaxone was changed to cefepime to cover *Pseudomonas*. Due to intractable agitation, she was transferred to the intensive care unit (ICU) for dexmedetomidine infusion.

On day three, gram-negative rods were detected in blood cultures. Antibiotics were changed to meropenem. After three days of antibiotics, a lumbar puncture was performed on day four, revealing 233 mg/dL of cerebrospinal fluid (CSF) protein, 117/ul of WBC, 36 mg/dL of glucose, negative cryptococcal antigen, negative enterovirus reverse transcription-polymerase chain reaction, Lyme immunoglobulin (Ig)M-IgG negative, and non-reactive venereal disease research laboratory test. The meningitis panel nucleic acid amplification test (NAT; the FilmArray® Meningitis/Encephalitis Panel, BioFire Diagnostics®, Salt Lake City, Utah) was positive for *H. influenza* (Table 1). Blood culture growth was also speciated as NTHi sensitive to ceftriaxone. CSF cultures showed no growth. Meropenem was changed to ceftriaxone, and the dexamethasone course was completed to four days. Repeat CT head with and without contrast was unremarkable.

**Table 1:** Meningitis panel nucleic acid amplification testing (the FilmArray® Meningitis/Encephalitis Panel)

Pathogens		Results
Escherichia coli K1		Negative
Hemophilus influenza		Positive
Listeria monocytogenes		Negative
Neisseria meningitidis		Negative
Streptococcus agalactiae		Negative
Streptococcus pneumoniae		Negative
Cytomegalovirus		Negative
Enterovirus		Negative
*Herpes simplex virus 1 *(HSV-1)		Negative
*Herpes simplex virus 2 *(HSV-2)		Negative
*Human herpesvirus 6 *(HHV-6)		Negative
Human Parechovirus		Negative
*Varicella-zoster virus *(VZV)		Negative
Cryptococcus neoformans/gattii		Negative

On day six, repeat blood cultures from day two were negative. After an improvement in her mental status, the patient stated that she started having an earache the day before admission, and two of her sisters also had ear infections recently. Her potential infectious nidus for the meningeal spread was likely otitis media, hypothesized due to her prior earache; however, her inpatient imaging studies failed to show any signs of mastoiditis or sinusitis. She fully recovered and was discharged on day 12. The ceftriaxone course was completed to 14 days after the first negative blood cultures.

## Discussion

pSS is a multisystemic, chronic autoimmune disease characterized by sicca complex and occasional extraglandular organ involvements [1]. It may be seen as a primary or a secondary entity associated with other rheumatological diseases, most commonly rheumatoid arthritis and systemic lupus erythematosus [2].

Increased infection risk is well known in autoimmune diseases [3,4] and is ascribed to primary disease processes as well as immunosuppressant and corticosteroid use [3,5]; however, pSS has differed with a lesser risk of community-acquired or opportunistic infections, hospital admissions, ICU admissions, and mortality [4-8], which was attributed to lesser use of aforementioned agents [8]. Unlike many agents that increase the risk of infections and admissions, HCQ use was shown to decrease the risk of infections likely due to its antimicrobial and immunomodulatory properties [5,8,9]. Increased infection incidence applied to both community-acquired and opportunistic infections [8,10]. Accordingly, infections were the co-dominant reasons for admission, with disease activity, in both inpatient [6] and intensive care units [11] in cases of connective tissue diseases in general and pSS specifically. Prevailing implicated infections were pneumonia, sepsis, skin and soft tissue infections, urinary tract infections, and opportunistic infections [10,12]. While central nervous system (CNS) infections did not have a high enough incidence to be mentioned separately, in one study, meningitis comprised 0.26% of all community-acquired infections leading to admissions in pSS patients, and meningitis incidence in pSS was not increased compared to the general population [13]. Risk factors for CNS infections were not separately reported; however, infectious risk factors in general for pSS patients were noted as older age, female sex, Caucasian ethnicity, and having a higher comorbidity burden. Serious infection rates among pSS patients requiring admissions have risen, with sepsis being increasingly more prominent [13].

*H. influenza* is a gram-negative rod that frequently colonizes the upper respiratory tract (URT) and is a major culprit of URT infections, pneumonia, and chronic lung disease exacerbations [14]. It has two distinct groups (typeable and non-typeable [NTHi]) based on the presence of a capsule. Encapsulated strains are serotyped based on their capsule polysaccharides (a-f) [15]. Even though NTHi commonly causes non-invasive infections of the URT (i.e., otitis media, sinusitis, and bronchitis), after the widespread *H. influenza* serotype b (Hib) vaccination since the late twentieth century, NTHi has become the most common pathogen of invasive *H. influenza* infections (i.e., epiglottitis, bacteremia, and meningitis) [12]. Vaccination against capsule antigen does not provide immunity against NTHi strains [16], and major host defense mechanisms against a first-time invasive NTHi infection include mucociliary clearance, barrier function, IgA1, complement activation, and phagocytosis [17,18].

Despite the rise in NTHi predominance of invasive *H. influenza* infections, NTHi remains a rare cause of purulent meningitides [19] and is more commonly seen in patients with predisposing factors (i.e., CSF leak and immunocompromised state) [20].

Even though our patient had pSS, she was only on HCQ, which was actually shown to decrease infection risk in rheumatological conditions. She did not have any other immunosuppressive conditions as a predisposing factor. Mucociliary clearance was mentioned [18] in protection against invasion of NTHi, and we hypothesize that impaired salivary or lacrimal glandular function may introduce an increased risk in pSS patients.

## Conclusions

This case underlines the importance of severe infectious complications regarding morbidity and mortality in pSS patients. These complications may occur even without immunosuppressive medication use and may be due to the primary disease pathophysiology. Special consideration is paramount in the selection of immunomodifying treatments in this group. Randomized clinical trials regarding the impact of different classes of therapeutic agents on patient morbidity/mortality and healthcare utilization are needed.
